# The Noblest Monument of the Queen's Reign

**Published:** 1887-12-31

**Authors:** Albert Rollit


### The Noblest Monument of the Queen's Reign.

By Sir Albert Rollit, M.P.

I desire to draw public attention to a very great and a very important object, for the welfare and interest of the metropolis in general, and of my own district, Islington, in particular. That object is a duty which appeals to the sympathy of everybody. From my point of view the appeal is essentially an appeal to Christians, but if there are some readers who do not attend places of worship I shall appeal to their religion of patriotism, their humanity, their duty to their fellow-men. Our God is not a selfish God but an unselfish God, and the highest religion, the truest religion which we can offer Him is service to our brother man. It matters not what religion we profess, our obligations in regard to the help of hospitals is the same. I am sorry to say that this combined movement is very necessary at the present time. I gather from papers which have been placed before me that there is a deficit in the hospital account of the Islington district alone of no less than £8,000. I regard the present very unfortunate state of our hospitals as a great blot in the history of our country, because if there be one feature which has marked the history of this century more than the history of any other, it has been the rise of those benevolent and philanthropic institutions that are so widely scattered throughout the length and breadth of our land. During the present reign they have increased most wonderfully, a testimony to the humanitarian spirit of the age. Think of the extraordinary progress. At the commencement of this century there were only fifty-one hospitals; now there are 496, or an increase of nearly tenfold. Then again we have the medical schools, the outcome of these great hospitals, and consequently the improved skill of the doctor who comes to see us in our own home. The result of all this has been that the death-rate of the United Kingdom has been materially reduced. This growth of philanthropy is eminently characteristic of this century, and it is one which we can contemplate with the greatest satisfaction during this period of Jubilee. Some people will tell you that the growth of a greater spirit of humanity is due to the French Revolution, and to similar causes, creating a spirit of brotherhood. But these glorious fruits of sympathy are, I think, due rather to our religious institutions and the devotion of ministers of religion to the service of the sick amongst them. Let us remember how they have brought to light cases of sickness and of suffering. Then let us remember also the brilliant sacrifices which the medical profession have made for the sick. I would not give a fig for the humanity of a man whose cat and dog were not the better for it, yet how many there are who never seem to remember the sufferings of their fellows or to care about them. Though we speak with pride and gratification of our medical institutions, there has unfortunately, from different causes, been a check upon their resources, and hence the object of this meeting is once more to enlist unsectarian support, to unite as many as possible in the good cause which has flourished so much in the past, and which we wish to make flourish in the future. It does not, I believe, need many arguments to induce you, who come here full of sympathy, to do what you can to induce other people who have not come here to enlist their sympathy in this great cause. I would mention one circumstance for the benefit of those who will not otherwise take an interest. They say, "Oh, these hospitals are so well established they will never be allowed to perish." But the case is not exactly thus. They would not be allowed to perish, for, if there were one thing more than another which would beget a revolution and give an impetus to Socialism, it would be the withdrawal of help from the suffering and afflicted poor. But if the funds are not forthcoming voluntarily our hospitals will not cease to exist, they will be supported through the rates. There may, perhaps, be some arguments in favour of such a principle, but there are more against it. Is it not a glorious sight to see against the walls of these institutions throughout the land the inscription, "Supported by voluntary contributions"? It indicates that humanitarian spirit which is most glorious when regarded in connexion with the history of our country. Let us draw upon the experience of other countries. In France, Germany, and other countries of Europe the hospitals are supported by a subsidised rate. In Paris the subsidy is no less than 3*s.* 6*d.* per inhabitant; in Rouen, 3*s.* 8*d.*; and in Leipsic, 4*s.* 8*d.* per inhabitant. Taxation is the alternative of the voluntary system. Then we must remember also that if you have taxation you have also State control and administration, which may be a greater evil still. I venture to think that if the hospitals were in the hands of the State their management as a whole would not be so economical as it is, while the lancets and the scalpels might not be so effective, judging from what we have recently heard about bayonets which have been supplied by the State. I trust, however, that when the needs of our hospitals are fully known there will be a material increase in the subscriptions; that we shall hear less of those people who will not do the Samaritan on any terms, and who say, "What I give is nothing to nobody." What they do they do secretly, and I do not suppose it is a great deal. Something, perhaps, like the case of the parson who said to his flock, "You gave nothing last year, and I, therefore, hope you will now double your contribution." I have thus far spoken in very general terms, but may I proceed to point out to you that our district has very strong claims? We have a deficiency of £8,000 a-year. The new hospital also is one in which all of us must, I am sure, take a deep interest; it will, I hope, be the brightest and best object of our local Jubilee commemoration. The Islington Dispensary—at the opening of which I deeply regret I was unable to be present—and the Nursing Institution have both struck me as having great claims upon the sympathies and the good will of the people. I trust we shall do all we can to extend this movement, if only for the benefit of those institutions which have made life so much more worth living. Again, let us remember the scientific skill which is gained from al the experience which is derived from human suffering—knowledge which finds its way into books and helps our medical men to cure sorrow in our households. That should be a reason for helping our hospitals, which even the richest ought not to be forgot. We have two recent striking illustrations—Sir Michael Hicks Beach, who is suffering from a malady where hospital-acquired knowledge will be of the highest possible advantage; and the case of the successor to one of the grandest empires of the history of the world—the Crown Prince of Germany, who, we hope, by the doctor's skill will be preserved to rule over a great and progressive people. The sufferings of the sick poor who seek our hospitals, form, as it were, the way by which our doctors derive their wonderful skill. This then is our argument, which must address itself to all who love health. We who are strong have this year held high festival in every quarter of our great City. But can any joy be compared to that which we feel when we know that we have helped to restore the sick man to health? Oh then let the *Jubilate* be said not only to the strong, but to the poor and suffering, and then our good Queen will say and feel that this Christian work of relieving our hospitals is the noblest monument of her reign.

